# Biodecolorization and Biodegradation of Methyl Red by Halophilic *Klebsiella aerogenes* WH2

**DOI:** 10.3390/microorganisms14040864

**Published:** 2026-04-11

**Authors:** Ruiping Wang, Haoxiong Li, Xiaoyi Ding, Yue Zhang, Zeyu Chen, Yiting Wang, Fangkui Wang, Yin Zhou

**Affiliations:** 1 Cooperative Innovation Center of Industrial Fermentation, Ministry of Education & Hubei Province, Hubei Key Laboratory of Industrial Microbiology, National “111” Center for Cellular Regulation and Molecular Pharmaceutics, Hubei University of Technology, Wuhan 430068, China; 2 National Key Laboratory of Agricultural Microbiology, Huazhong Agricultural University, Wuhan 430070, China

**Keywords:** methyl red, biodegradation, *Klebsiella aerogenes*, halophile

## Abstract

The textile industry wastewater contaminated by azo dyes usually contains a certain amount of salinity. Therefore, screening for microorganisms capable of degrading azo dyes in saline environments is of great significance. In this study, the decolorizing activity of azo dye methyl red (MR) by *Klebsiella aerogenes* WH2 (WH2), newly isolated from soil, was evaluated. WH2 was able to decolorize 92.4% and 86.0% of MR at concentrations of 200 mg/L and 300 mg/L within 24 h, respectively. Given that WH2 exhibited enhanced growth and superior degradation capacity in the presence of 2.5% NaCl compared to salt-free conditions, it can be classified as a slight halophile. Approximately 87.7% of MR was removed by WH2 in the presence of 10.0% NaCl within 24 h. Azoreductase activity assays indicated that WH2 retained higher enzyme activity in the presence of NaCl concentrations not exceeding 7.5%. The degradation products and putative metabolic pathways for MR degradation by WH2 were analyzed using FTIR and LC-MS. Phytotoxicity analysis based on seed germination of *Vigna radiata* indicated that the degradation products of MR exhibited less toxicity than the parent compound. The high degradation efficiency of MR under high salt concentrations makes WH2 a promising candidate for the treatment of saline textile wastewater.

## 1. Introduction

Azo dyes are a kind of synthetic dyes that are characterized by the azo group (–N=N–) coupled with two symmetrical and/or asymmetrical identical or non-azo alkyl or aryl radicals [1]. Azo dyes are widely used in textiles, leather, plastics, cosmetics, and food processing industries [2]. However, inefficient dyeing processes in textile industry can lose 20–50% of dyes, which are discharged directly into wastewater and ultimately pollute the environment [2]. The effluents containing azo dyes and their metabolites contaminate groundwater and disrupt aquatic ecosystems, leading to eutrophication and ecological imbalance. These azo dyes also inhibit the growth and germination of ecologically important biomasses, causing soil erosion, fertility loss, and harm to wildlife. In addition, many azo dyes are toxic, carcinogenic, and mutagenic, and they can pose a threat to human health. Thus, the removal of azo dyes from textile effluents has been a major concern [3].

It is necessary to develop effective removal methods to treat wastewater containing azo dyes. Several physical and chemical techniques have been applied to eliminate azo dyes from wastewater. Absorption is considered to be the simplest and fastest method to remove azo dyes from wastewater. Carbon adsorbent prepared from fennel (*Foeniculum vulgare*) seeds, bark of the *Dodonaea viscosa* (Hopbush) plant, sodium carbonate-treated jute fiber (SCTJF), and other materials displayed the ability to remove methyl red from wastewater [4,5,6]. Benzene can be used as the extractant to remove methyl red from its aqueous solution by liquid–liquid extraction [7]. However, the preparation processes of MR absorbents are complex, and the removal process of MR requires other chemicals, which may cause secondary pollution. Compared to physicochemical methods, biological degradation methods are more economical and environmentally friendly. Previous research showed that many types of microorganisms, including bacteria, fungi, algae, and yeast, have degradation abilities for azo dyes. Several pure bacterial species have been identified with decolorization abilities of textile effluent containing azo dyes, such as *Pseudomonas luteola*, *Bacillus cereus*, *Bacillus subtilis*, *Aeromonas hydrophila*, *Proteus mirabilis*, and *Rhizobium radiobacter* [3]. Microbial consortia possess broader enzymatic capacity and higher degradation efficiency than pure strains, making them widely used in biodegradation studies at both laboratory and industrial scales. Several microbial oxidoreductase enzymes, such as azoreductase, laccase, manganese peroxidase, and lignin peroxidase, play important roles in the microbial azo dye decolorization process [8].

Methyl red (MR), an anionic azo dye, is widely used in printing and textile dyeing industries due to its strong color-fixing performance and mild fading [9]. In addition, MR is used as an acid–base indicator in laboratories. MR displays red color in acidic solutions having a pH of 4.4, orange color in the solutions having a pH range of 4.4–6.2, and yellow color in solutions having a pH higher than 6.2 [10]. Several microbial strains have been found to possess the ability to degrade methyl red. For example, *Schizophyllum commune* 15R-5-F01 showed the ability to degrade over 96% of MR (100 mg/L) within 3 h [11]. *Sphingomonas paucimobilis* decolorized 99.63% of MR (750 ppm) within 10 h at 30 °C and pH 9 [12]. *Kocuria indica* DP-K7 degraded 68% of MR (200 μM) after 160 h of incubation [13]. *Aspergillus versicolor* decolorized 79.64% of MR (100 mg/L) after incubation [14].

The textile industry wastewater contaminated by azo dyes usually contains a certain amount of salinity (0.1–10.0%), which is used for dye fixation [15]. However, salts in wastewater affect microbial growth, thereby affecting the biodegradation efficiency of conventional microorganisms [15]. Hence, it is necessary to screen the strain that retains azo dye-degrading activity in the presence of high salt concentrations. In this study, a strain of *Klebsiella aerogenes* WH2 (hereinafter WH2) was isolated, and its decolorization ability on MR was investigated. The effects of physicochemical parameters on MR decolorization efficiency by WH2 were observed. The effect of NaCl on degradation efficiency, the growth, and azoreductase activity of WH2 was investigated. The biodegradation products of MR treated by WH2 were analyzed by FTIR and LC-MS. The toxicity of degradation products of MR treated by WH2 was determined on the seed germination of mung bean (*Vigna radiata*). This study could contribute to the consideration of *Klebsiella aerogenes* WH2 in the bioremediation of dye-contaminated wastewater with a higher concentration of salt.

## 2. Materials and Methods

### 2.1. Dye and Media

The textile azo dye methyl red was purchased from Shanghai Yuanye Biotechnology Co., Ltd. (Shanghai, China). Luria–Bertani (LB) medium contains 10.0 g/L tryptone, 5.0 g/L yeast extract, and 10.0 g/L NaCl. The experimental medium I (MI) contains 5.1 g/L K_2_HPO_4_, 3.0 g/L KH_2_PO_4_, 0.5 g/L NaCl, 2.0 g/L glucose, and 1.0 g/L yeast extract. The experimental medium II (MII) contains 5.1 g/L K_2_HPO_4_, 3.0 g/L KH_2_PO_4_, and 0.5 g/L NaCl.

### 2.2. Strain Isolation and Identification

Soil samples were collected from Shizishan, Wuhan, China. The MR-degrading bacteria were isolated from a soil sample using the selective enrichment method described previously, with some modifications [16]. A total of 5 g of each sample was diluted with 0.9% physiological saline and inoculated on the ligninolytic selection medium (NaNO_3_ 2.5 g/L, KH_2_PO_4_ 1.0 g/L, MgSO_4_ 1.0 g/L, NaCl 1.0 g/L, CaCl_2_ 0.5 g/L, alkali lignin 5 g/L, and yeast extract 0.1 g/L). The strain, WH2, was isolated with the ability to decolorize aniline blue. The genomic DNA of WH2 was extracted by the boiling method, and the 16S rDNA gene of the bacteria was amplified by PCR with universal bacterial primers 27F and 1492R. The PCR products were sent for sequencing, and the 16S rRNA sequence was submitted to NCBI under the accession number of PZ071976. Phylogenetic trees were constructed by using 16S rRNA gene sequences of WH2 and other closely related species by the neighbor-joining method with 1000 bootstrap replicates using the MEGA 11 software package. *Escherichia coli* ATCC 1775 and *Escherichia fergusonii* ATCC 35469 were used as the outgroup. Bootstrap values ≥50% were shown at the nodes.

### 2.3. The Effect of MR and NaCl on the Growth of WH2

To investigate the effect of MR on the growth of WH2, WH2 cells were grown in MI or MII medium (containing 200 mg/L MR). To study the effect of MR concentrations on the growth of WH2, WH2 cells were grown in MI medium supplemented with different concentrations of MR (100, 200, and 300 mg/L). To investigate the effect of NaCl concentration on the growth of WH2, WH2 cells were grown in MI medium (containing 200 mg/L MR) with different initial concentrations of NaCl (0, 2.5%, 5.0%, and 7.5%). The bacterial cultures were incubated at pH 7.0, 37 °C, and 200 rpm. Samples were collected at different time points, and the absorbance was measured at OD _600nm_.

### 2.4. MR Decolorizing Ability Analysis

Stock solution of MR (5000 mg/L) was prepared by dissolving 0.5 g MR in 100 mL of 95% ethanol. The strain WH2 was grown overnight at 37 °C in LB medium, and the cultures were diluted to a final OD_600_ of 0.8 in LB medium and inoculated into MI medium containing MR (200 mg/L) at 10% (*v*/*v*) inoculum. Sample aliquots were collected and centrifuged after incubation at 37 °C for 0, 3, 6, 9, 12, and 24 h, respectively. The clear supernatant of different MR samples treated by WH2 was used to record absorbance using a spectrophotometer at 428 nm [17]. The medium containing the same concentration of MR but without the inoculation of WH2 was used as a control. All experiments were performed in triplicate.

Decolorization percentage was calculated using the following formula:

(1) $$Decolorization\;\left(\%\right)=\;\frac{Initial\;absorbance\;-\;Final\;absorbance}{Initial\;absorbance}\times \;100\%$$

When studying the effect of a certain parameter on MR decolorization, all other variables were kept constant as described previously. To study the effect of initial dye concentration on MR decolorization, the decolorization assays were performed under different initial MR concentrations (100, 200, 300, and 500 mg/L). To study the effect of temperature on MR decolorization, the decolorization assays were performed under different temperatures (30, 37, 45, and 50 °C). This experiment was carried out in a water bath. To study the effect of pH on MR decolorization, the decolorization assays were performed under different pH values (7, 8, 9, and 10). To study the effect of inoculum size on MR decolorization, the decolorization assays were performed under different inoculum sizes of *Klebsiella aerogenes* WH2 (1%, 5%, 10%, and 15% (*v*/*v*)). To study the effect of carbon and nitrogen sources on MR decolorization, the decolorization assays were performed in MII media with different carbon and nitrogen sources. Fructose, glucose, starch, lactose, and sucrose at a concentration of 0.2% (*w*/*v*) were added separately to MII medium to study the effect of carbon sources on degradation. Yeast extract, urea, and ammonium sulfate at a concentration of 0.1% (*w*/*v*) were added separately to MII medium to study the effect of nitrogen source on degradation. To study the effect of salinity concentration on MR decolorization, the decolorization assays were performed in MI media with different NaCl concentrations (0, 2.5%, 5.0%, 7.5%, and 10.0%). To study the effect of metal ions on MR decolorization, the decolorization assays were performed in MII media with 1 mM of CuCl_2_, NiCl_2_, CoCl_2_, CaCl_2_, FeSO_4_, MgCl_2_, CdCl_2_, and MnCl_2_, respectively.

### 2.5. Enzyme Analysis

To investigate the effect of initial dye concentration on azoreductase activity, WH2 cells were grown in LB medium (containing 2.5% NaCl) supplemented with different concentrations of MR (0, 50, and 100 mg/L). Cells were harvested by centrifugation for 2 h and 4 h. To study the effect of initial salt concentration on azoreductase activity, WH2 cells were grown in LB medium (containing 100 mg/L MR) supplemented with different concentrations of NaCl (0, 2.5%, 5.0%, 7.5%, 10.0%, and 12.5%). Cells were harvested by centrifugation for 2 h and 4 h. The above bacterial cells were collected by centrifugation, washed twice, and resuspended in 20 mM potassium phosphate buffer (pH 7.0). The cells were then disrupted using an ultrasonic homogenizer JY 92-IIN (Scientz Co., Ltd., Ningbo, China). The protein concentration in the supernatant was determined using the Bradford method with bovine serum albumin (BSA) as the standard. After measuring the protein concentrations, the concentrations were normalized to a consistent level. The azoreductase activity was determined by monitoring the absorbance decrease at 430 nm in a 3 mL reaction mixture containing 50 μM methyl red (MR) and 100 μM NADH in potassium phosphate buffer (20 mM, pH 7.0).

### 2.6. UV-Vis and Fourier-Transform Infrared (FTIR) Spectroscopy

The UV-vis spectral analysis of supernatants was performed by a NanoDrop One UV–Vis spectrophotometer (Thermo Scientific, Waltham, MA, USA) in the range of 300–600 nm. FTIR analysis was performed as described previously, with some modifications [18]. The supernatant of MR was obtained in the same way as described previously. Equal volumes of chromatographic-grade ethyl acetate (HPLC grade) were added to the supernate for extraction. The separated extract was concentrated at 45 °C with a rotary evaporator (RE-52, Yarong Biochemical Instrument Co., Ltd., Shanghai, China), and anhydrous sodium sulfate was added for dehydration to obtain a dry substance. The dry substance was dissolved in methanol (HPLC grade). The FTIR spectra of the samples before and after MR degradation were acquired on a Nicolet iN10 FTIR spectrometer equipped with an ATR accessory (Thermo Fisher Scientific Co., Ltd., USA) over the range of 4000–400 cm^−1^.

### 2.7. Liquid Chromatography-Mass Spectrometry (LC–MS)

The biodegradation products of MR were determined by liquid chromatography-mass spectrometry (LC–MS), as described previously, with some modifications [18]. The supernatant of MR was extracted and concentrated as described in the FTIR analysis. The dry substance was dissolved in ethyl acetate (HPLC grade). The samples were filtrated and sterilized by a 0.22 μm microporous membrane filter. A certain amount of sample (10 μL) was injected into the C18 column (50 × 2.1 nm, 2.1 μm), and the metabolites of MR before and after biodegradation were detected by LC-MS (Q Exactive, Thermo Fisher Scientific, USA). Mobile phase A was an aqueous formic acid solution (0.1%), while mobile phase B was a solution with methanol and formic acid (0.1%). Chromatographic separation was achieved by gradient elution. Electrospray Ionization (ESI) conditions were set as follows: capillary voltage = 3500 V; capillary temperature = 350 °C; and drying temperature = 300 °C. The sheath gas and auxiliary gas flow rates were 35 and 10 Arb, respectively. The primary spectra were collected using full-scan MS analysis in a mass range of *m*/*z* 50–700.

### 2.8. Toxicity of Degradation Product of MR

The phytotoxicity of the degradation metabolite of MR (100 mg/L) was evaluated on the seed germination of mung bean (*Vigna radiata*). The seeds of the mung bean (*Vigna radiata*) were obtained from the local marketplace in Wuhan, China. The seeds of *V. radiata* were sterilized with 70% ethanol for 1 min, and sodium hypochlorite solution (2%) for 10 min, and this was followed by a rinse with sterile water. The supernatant of MR and MR treated by WH2 for 24 h was diluted to 50% before applying to the seeds of *V. radiata*. Surface-sterilized seeds of *V. radiata* were kept aseptically on autoclaved blotting papers in each sterile Petri plate. Ten milliliters of MR (100 mg/L, diluted to 50%) or MR treated by WH2 was supplemented over the individual plate containing *V. radiata* seeds. Distilled water was supplemented to the control plate, and all plates were incubated at 25 °C for 6 days under dark conditions. The experiment was replicated three times. The toxicity effect was measured in terms of percent germination and the lengths of root, stem, and leaves.

### 2.9. Statistical Analysis

SPSS 23.0 software (SPSS Inc., Chicago, IL, USA) was used for statistical analysis to analyze the significant differences between different experimental groups in the effect of metal ions, carbon source, and nitrogen source on MR decolorization, and phytotoxicity studies of metabolites. Data were analyzed using one-way ANOVA and Duncan test (*p* < 0.05) followed by the least significant difference (LSD) test. Data are presented as mean ± standard deviation (M ± SD).

## 3. Results and Discussion

### 3.1. Isolation and Identification of Klebsiella aerogenes WH2

**Figure 1:**
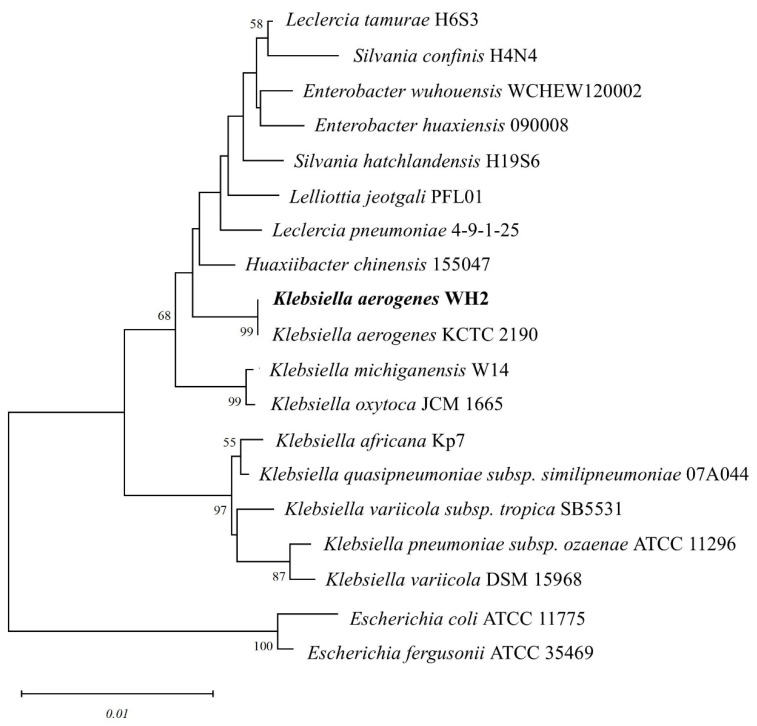
Phylogenetic tree of *Klebsiella aerogenes* WH2 and other 16 closely related species. *Escherichia coli* ATCC 1775 and *Escherichia fergusonii* ATCC 35469 were used as outgroups. Numbers at nodes indicate the percentage of bootstrap support based on 1000 bootstrap replicates under neighbor-joining analysis. Only bootstrap values above 50% were shown. Bar 0.01% substitution per site.

The isolate, *Klebsiella aerogenes* WH2, had a characteristic white appearance on the LB medium. The 16S rRNA gene sequence of WH2 was submitted to the NCBI database (PZ4071976), and the BLAST results indicated that WH2 shared 100% 16S rRNA sequence identity with *Klebsiella aerogenes*. A maximum likelihood phylogenetic tree was constructed based on 16 type strains that are closely related to strain WH2 (Figure 1). Based on the phylogenetic tree analysis, the strain WH2 was most closely related to *Klebsiella aerogenes* KCTC 2190 (Figure 1). Both BLAST and phylogenetic tree analyses indicated that WH2 was most closely related to *Klebsiella aerogenes*; accordingly, the strain WH2 was designated *Klebsiella aerogenes* WH2 in this study (hereafter referred to as WH2). Thus, the study of the degradation ability of MR by *Klebsiella aerogenes* WH2 further strengthens the potential of the strain for use in environmental pollutant degradation.

### 3.2. MR Decolorization Ability of Klebsiella aerogenes WH2

**Figure 2:**
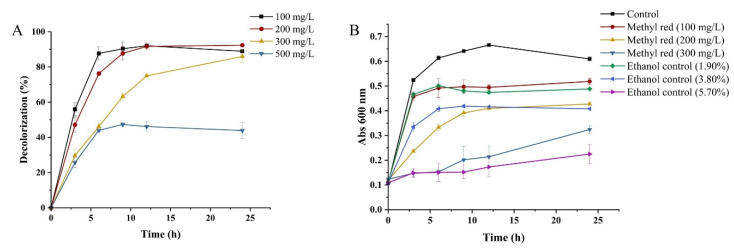
Decolorization ability of MR by WH2. (**A**) The decolorization ability of different concentrations of MR by WH2; (**B**) the growth curve of WH2 under different concentrations of MR. The ethanol control (1.90%), ethanol control (3.80%), and ethanol control (5.70%) correspond to the concentration of ethanol present in 100 mg/L, 200 mg/L, and 300 mg/L of MR, respectively.

The decolorization efficiency of WH2 in the presence of 100–500 mg/L of MR was studied. As shown in Figure 2A, the decolorization rates of MR at 100 mg/L, 200 mg/L, and 300 mg/L after the treatment by WH2 after 6 h were 87.7%, 76.3%, and 46.2%, respectively. The decolorization rates of MR at 100 mg/L, 200 mg/L, and 300 mg/L by WH2 after 24 h of treatment were 88.9%, 92.4%, and 86.0%, respectively. However, the decolorization rates of MR at 500 mg/L after the treatment of WH2 for 6 and 24 h were only 44.0% and 43.9%, respectively. Thus, WH2 exhibited higher decolorization activity toward MR at concentrations less than or equal to 300 mg/L, but showed lower decolorization ability toward MR at 500 mg/L. The reason might be that higher concentrations of azo dye are toxic to bacterial growth and might inhibit the enzyme activity of microorganisms [8]. The decolorization activity of microorganisms on azo dyes varied based on the different microorganisms and the structure of the azo dyes. Based on the previous literature, different microorganisms showed the ability to decolorize MR at concentrations between 20 and 500 mg/L [11,12,13,14]. Compared with previous studies, WH2 was capable of decolorizing MR at relatively higher concentrations.

The effect of MR on the growth of *Klebsiella aerogenes* WH2 was investigated. As shown in Figure 2B, with increasing concentrations of MR, the inhibitory effect of MR on the growth of WH2 became more pronounced. Due to the low solubility of methyl red in water, the MR solution was solubilized in ethanol. To rule out the effect of ethanol on WH2 growth, experiments were conducted simultaneously to assess the impact of ethanol concentrations equivalent to those present in the MR solutions on WH2 growth. The results showed that 100 mg/L of MR and the corresponding ethanol (1.90%), 200 mg/L of MR and the corresponding ethanol (3.80%), and 300 mg/L of MR and the corresponding ethanol (5.70%) exhibited similar inhibitory effects on the growth of WH2, suggesting that the growth inhibition caused by MR may be attributed to ethanol. The previous literature indicates that the MIC and MBC values (%) of ethanol on 57 isolates of *Klebsiella pneumoniae* were 0.44–0.30% and 4.4–0.6%, respectively [19]. Therefore, the inhibitory effect of high-concentration MR on the growth of MR may be primarily attributed to the inhibitory effect of ethanol.

### 3.3. Effect of Environmental Factors on MR Decolorization

The decolorization efficiency of dyes is strongly affected by several environmental factors, including inoculum size, temperature, pH, metal ions, carbon source, and nitrogen source. In the present study, the effects of all these environmental factors on the MR decolorization potential of strain WH2 were evaluated.

**Figure 3:**
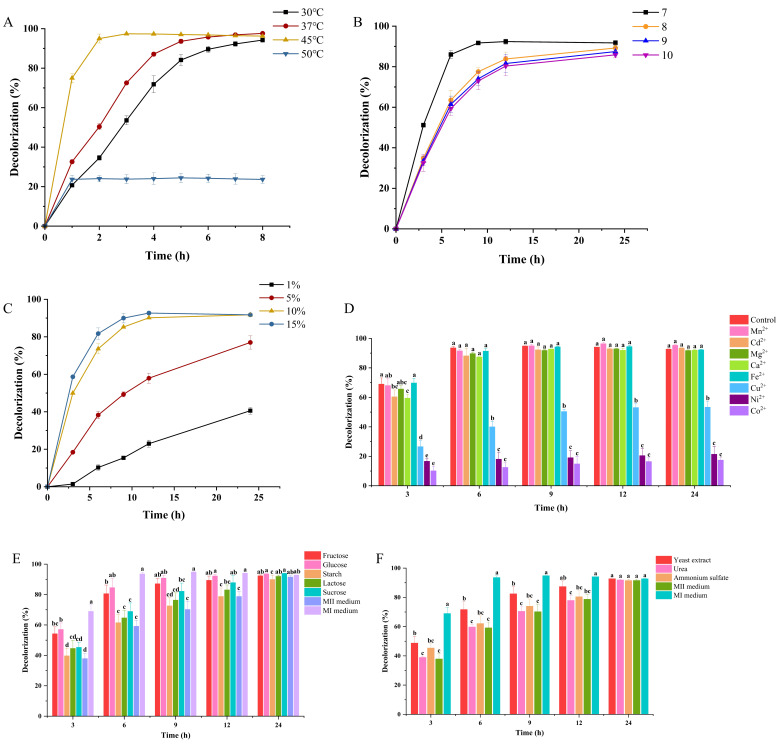
The effects of temperature (**A**), pH (**B**), inoculum size (**C**), metal ions (**D**), carbon source (**E**), and nitrogen source (**F**) on the decolorization efficiency of MR by WH2. Values are means of triplicate experiments, and error bars represent standard deviations calculated from three biological replicates. Different lowercase letters indicate significant differences (*p* < 0.05) among different metal ions, carbon or nitrogen sources at the same treatment time.

Due to the influence on bacterial growth and enzyme activities, temperature has an apparent effect on bacterial decolorization abilities on azo dyes. The effect of temperature on MR decolorization by WH2 was evaluated (Figure 3A). The decolorization rates of the MR by WH2 ranged from 94.2% to 97.6% after treatment for 8 h within the temperature range of 30–45 °C. However, the decolorization rate decreased to 23.7% after treatment for 8 h at a temperature of 50 °C. The decolorization rates of MR at 45 °C were highest among all the temperatures after treatment for 1 to 6 h, and the decolorization rate of MR reached 75.0% after treatment for 1 h at 45 °C. Therefore, the optimal decolorization temperature of MR by WH2 is 45 °C. This result was consistent with those reported in the earlier literature that *Bacillus subtilis* azo reductase AzoR1 exhibited high degradation abilities on azobenzene, methyl red, Orange G, and Congo Red dye at 45 °C [20]. The optimal decolorization temperature is related to the optimal temperature of degradation enzymes in microorganisms, especially azo reductase, which is the dominant enzyme involved in the degradation of azo dyes [21]. The decrease in decolorization activity of MR at 50 °C can be attributed to the loss of cell viability or the denaturation of degradation enzymes, such as azo reductase [21]. Actual textile effluent usually has the property of a wide range of temperatures (30–55 °C) [22]. Thus, the bacterium WH2 exhibited higher decolorization activity between 30 and 45 °C. This result is consistent with the previous literature that found that the optimum temperature for the bacteria to remove the toxic dyes ranges from 30 to 45 °C [23].

The pH is one of the important factors that affect the growth of microorganisms and the dye decolorization process. The effect of pH on the decolorization efficiency of MR by WH2 was evaluated (Figure 3B). It can be seen that the decolorization rate of MR by WH2 at pH 7 was higher than the decolorization rates under other pH conditions at different treatment times. The decolorization rates of MR (200 mg/L) were 86.0% and 91.7% after the treatment by WH2 for 6 and 24 h at pH 7, respectively. The decolorization rates were 89.2%, 87.5%, and 85.9% after treatment for 24 h at pH 8, 9, and 10, respectively. Thus, the optimal pH for the decolorization of MR by WH2 was 7. The optimum pH for decolorization of MR was also 7 by some other bacteria, such as *Anoxybacillus* sp. PDR2, actinobacterium *Zhihengliuella* sp. ISTPL4 [18,24]. Some other bacteria, such as *Sphingomonas paucimobilis* and *Pseudomonas aeruginosa*, had the best decolorization effect on MR when the pH was 9 [12,25]. Based on the previous literature, the optimal pH value for dye degradation by bacteria usually ranges from 6 to 10 [26]. The difference in decolorization abilities for MR by different bacteria might be that the activities of enzymes for dye degradation are affected by the pH in the medium [27].

The inoculum size of microorganisms has a great influence on dye biodecolorization. The effect of the inoculum size of WH2 on the decolorization efficiency of MR was investigated. As shown in Figure 3C, the removal efficiency of the MR by WH2 increased with the increase in inoculum size under the same bacterial treatment time. When the inoculum size is 1% (*v*/*v*), the decolorization rate increased slowly within 6 h, and reached 40.6% at 24 h of treatment. When the inoculum sizes of WH2 were 5% (*v*/*v*), 10% (*v*/*v*), and 15% (*v*/*v*), the decolorization rates of MR at 24 h were 77.0%, 91.7%, and 91.7%, respectively. The decolorization rate was higher when the inoculum sizes were 10% (*v*/*v*) and 15% (*v*/*v*). The decolorization capacity increased with the increase in the initial inoculum size, and the reason might be that higher amounts of bacteria in the medium can secrete more enzymes and thus enhance the decolorization process. Junnarkar et al. reported that the decolorization rate of Direct Red 81 by the bacterial consortium increased with an increase in the inoculum size, reaching a maximum at 20% (*v*/*v*) inoculum size, but the decolorization rate did not vary significantly when the inoculum size was beyond 20% (*v*/*v*) [28]. Given the negligible difference between the degradation efficiencies using 10% (*v*/*v*) and 15% (*v*/*v*) inoculum, 10% (*v*/*v*) inoculum was adopted for the following experiments.

Textile effluent contains dyes and a large amount of heavy metal ions, such as Cr (VI), Cd (II), Pb (II), and Zn (II) [29]. High concentrations of metal ions are toxic to microorganisms. The effect of different metal ions on the decolorization abilities of WH2 on MR was shown in Figure 3D. After the treatment by WH2 for 24 h, the decolorization rate of MR exceeded 90% at the presence of 1 mM of Ca^2+^, Fe^2+^, Cd^2+^, Mg^2+^, and Mn^2+^, and the decolorization rate exceeded 50% at the presence of 1 mM of Cu^2+^. However, Ni^2+^ and Co^2+^ greatly reduced the decolorization rate of MR to 21.5% and 17.5% after treatment by WH2 for 24 h, respectively. The previous literature indicated that some metal ions (i.e., Cu^2+^, Ni^2+^, Co^2+^, Cd^2+^, Pb^2+^, and Cr^3+^) at 1 mM concentration decreased the decolorization rate on MR by *Bacillus* sp. strain UN2 [30]. In contrast to the literature, this study differed in that WH2 still exhibited decolorization ability in the presence of Cd^2+^ and Cu^2+^. The mechanism for the reduction in decolorization efficiency by heavy metal ions may be attributed to the inhibition of the laccase activity by binding to the enzyme’s active sites or causing structural changes [31]. Thus, it can be speculated that the inhibition of Ni^2+^ and Co^2+^ on the decolorization of MR may be caused by the inhibition of the laccase activity that is used for MR degradation. Possessing MR decolorization abilities under metal ion conditions, such as Cd^2+^ and Cu^2+^, endows WH2 with the potential to treat metal ion-rich textile effluent.

Carbon and nitrogen sources can be utilized by microbes for their growth and metabolism to produce enzymes for azo dye decolorization, and thus they can influence biodegradation efficiency [8]. The effects of carbon and nitrogen sources on the decolorization efficiency of MR by WH2 were investigated. The decolorization rates of MR under different carbon sources are exhibited in Figure 3E. After treatment by WH2 for 3 and 6 h, the decolorization rate of MR by WH2 in MI medium was significantly higher than that in MII medium with glucose or fructose as the carbon source, with the latter being significantly higher than that in MII medium with other carbon sources in MII medium or only MII medium (*p* < 0.05). The decolorization rates of MR under different nitrogen sources are exhibited in Figure 3F. After treatment by WH2 for 3, 6, and 9 h, the decolorization rate of MR by WH2 in MI medium was significantly higher than that in MII medium with yeast extract as the nitrogen source, with the latter being significantly higher than that in MII medium with other nitrogen sources in MII medium or only MII medium (*p* < 0.05). However, there was no significant difference among the decolorization rate of MR in MI medium and MII medium after treatment by WH2 for 24 h. The initiation of decolorization activity of MR by WH2 required the presence of both carbon and nitrogen sources. Deprivation of either nutrient led to a significant decrease in the decolorization rate, and concurrent deficiency of both yields the minimal decolorization performance. Additionally, fructose and glucose were identified as the optimal carbon sources, and yeast extract as the optimal nitrogen source, demonstrating markedly higher decolorization activity than their counterparts.

Some microorganisms utilize azo dyes as the sole source of carbon and nitrogen for growth and metabolism [8]. *Kocuria indica* DP-K7 can utilize 200 μM MR as the sole carbon source both in SEM agar and liquid broth [13]. Previous results demonstrated that the growth of WH2 was inhibited by MR (Figure 2B). To understand why carbon and nitrogen sources affected the decolorization rate of the MR by WH2, the growth curves of WH2 were measured and analyzed. As shown in Figure S1, during the initial 12 h of cultivation, the growth of WH2 was more pronounced in the medium without MR compared to the medium containing MR, regardless of whether MI medium or MII medium was used. This indicated that MR inhibits the growth of WH2 during cultivation. WH2 showed improved growth in MI medium (containing glucose and yeast extract) compared to MII medium (no carbon and nitrogen source). In the presence of MR, WH2 exhibited better growth in MI medium than in MII medium. This observation also explains why WH2 displayed higher decolorization activity in the carbon/nitrogen-supplemented MI medium compared to the carbon/nitrogen-free MII medium.

### 3.4. Effect of NaCl Concentration on MR Decolorization

The wastewater contaminated by azo dyes usually contains a certain amount of salinity (0.1–10.0%), which is used for dye fixation [15]. However, salts in wastewater affect microbial growth, thereby affecting the biodegradation efficiency of conventional microorganisms [15]. Hence, it is necessary to determine the effect of salinity concentration on the decolorization efficiency of MR by WH2.

**Figure 4:**
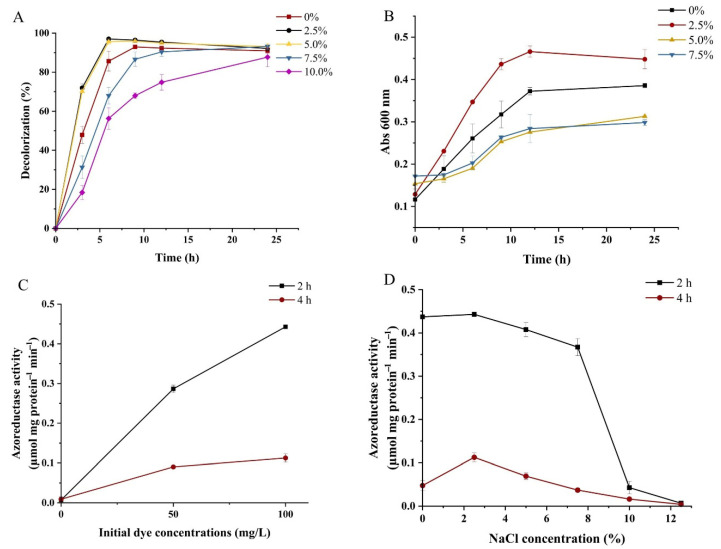
The effects of NaCl concentration on the decolorization efficiency, the growth, and azoreductase activity of WH2. (**A**) The effects of NaCl concentrations (2.5%, 5.0%, 7.5%, and 10.0%) on the decolorization efficiency of MR by WH2; (**B**) the effects of NaCl concentration (2.5%, 5.0%, and 7.5%) on the growth of WH2; (**C**) the effect of MR (50 and 100 mg/L) on the azoreductase activity of WH2 after treatment for 2 and 4 h; (**D**) the effect of NaCl concentration (2.5%, 5.0%, 7.5%, 10.0%, and 12.5%) on the azoreductase activity of WH2 after treatment for 2 and 4 h.

As shown in Figure 4A, around 71.9% and 70.2% MR was removed in the presence of 2.5% and 5% NaCl in 3 h, respectively. However, only 47.9% MR was removed under NaCl-free conditions in 3 h. Although a further increase in NaCl concentration resulted in significant inhibitory effects on decolorization, WH2 could still remove 31.3% and 18.4% MR within 3 h in the presence of 7.5% and 10% NaCl, respectively. The differences among all of the experimental groups gradually diminished after treatment by WH2 for 12 h, and the degradation rates in all experimental groups were above 87.7% after the treatment for 24 h. The decolorization efficiencies of MR by WH2 increased when NaCl concentrations were at 2.5% and 5%, but the decolorization rates decreased when NaCl concentrations exceeded 7.5%. Some microorganisms are sensitive to high salt concentrations, while other bacteria still display degradation abilities on azo dyes at high concentrations of salt solution [26]. *Lactiplantibacillus plantarum* SS-AU1 degraded 94.8% of MR (100 mg/L) within 24 h in the presence of 12% NaCl [32]. Thus, the results revealed that WH2 exhibited decolorization ability of MR in high-salinity conditions.

### 3.5. Effect of NaCl Concentration on the Growth of WH2

Lower NaCl concentrations (2.5% and 5%) enhanced MR degradation, which may be related to the influence of NaCl concentration on the growth of WH2. Thus, a subsequent investigation was conducted to examine the effect of NaCl on the growth of WH2. The results of growth curves of WH2 at various salt concentrations (0–7.5% *w*/*v*) are shown in Figure 4B. WH2 exhibited enhanced growth in medium with 2.5% NaCl compared to medium without NaCl. However, higher NaCl concentrations (5% and 7.5%) inhibited the growth of WH2 compared to the medium without NaCl. As seen, the optimized salt concentration for WH2 was 2.5%. Based on the previous literature, slight halophiles grow optimally at 0.2–0.85M (1–5%) sodium chloride (NaCl). In contrast, nonhalophiles grow optimally in less than 0.2M (1.17%) NaCl conditions [33]. Therefore, WH2 can be considered a slight halophile. In this study, the differences in decolorization efficiency under different NaCl concentrations were primarily attributed to the inhibitory or promoting effects of salinity on the growth of WH2. At unsuitable NaCl concentrations, the growth rate of WH2 decreased significantly, leading to slow biomass accumulation, which in turn weakened the overall decolorization efficiency on MR. This indicated that NaCl concentration indirectly affected the decolorization efficiency of WH2 on MR by regulating their proliferation rate.

### 3.6. Effect of NaCl Concentration on Azoreductase Activity

The enzymes involved in the bacterial degradation of azo dyes include Azoreductase, Laccases, Manganese peroxidase (MnP), Lignin peroxidase (LiP), Polyphenol oxidase (PPO), etc. Azoreductases represent a major class of enzymes produced by azo dye-degrading bacteria and fungi, facilitating the decolorization and degradation of these compounds [34]. Previous studies have shown that the production of azoreductase can be induced by methyl red [35,36]. The effect of MR concentration on the azoreductase activity was further investigated. As shown in Figure 4C, the results indicated that 50 mg/L of MR induced the production of azoreductase, and azoreductase activity increased as the MR concentration increased to 100 mg/L. However, the azoreductase activities decreased after the addition of MR for 4 h. The azoreductase activities were also investigated after the addition of MR for 2 h in other literature [37]. The reason might be that after the treatment for 2 h, the concentration of MR decreased. Thus, the need for high azoreductase activity decreased, leading to a gradual decline in azoreductase activity.

WH2 exhibited higher degradation activity at 2.5% NaCl compared to the absence of NaCl. To further investigate the specific reasons for the effect of NaCl concentration on the degradation of MR by WH2, the azoreductase activities of WH2 were measured in media containing NaCl ranging from 0 to 12.5% in the presence of MR. As shown in Figure 4D, the results demonstrated that azoreductase activity at 2.5% NaCl was similar to that without NaCl after the treatment by WH2 for 2 h. As NaCl concentration increased, azoreductase activity slightly decreased, but relatively high activity was maintained up to a NaCl concentration of 7.5%. Azoreductase activity dropped sharply starting from a NaCl concentration of 10%. This trend was consistent with previous studies that a reduction in azoreductase activity was observed under elevated salt conditions [38]. Although an appropriate concentration of NaCl may promote microbial metabolism, enzyme structures can be disrupted when salinity exceeds a certain threshold, leading to decreased enzyme activity. High NaCl conditions increase osmotic pressure, potentially inducing plasmolysis, impairing metabolic enzyme function, and inhibiting microbial growth [39].

In this study, the azoreductase of WH2 maintained high activity at NaCl concentrations not higher than 7.5% after treatment for 2 h. This result was consistent with the previous results that WH2 maintained decolorization activity in the presence of no higher than 10.0% of NaCl. The optimal azoreductase activity was observed at 0 and 2.5% NaCl, whereas optimal degradation occurred at 2.5% and 5% NaCl, and optimal growth of WH2 was at 2.5% NaCl. The discrepancy between optimal azoreductase activity, optimal degradation efficiency, and optimal growth may be explained by the fact that azoreductase activity at 0 and 2.5% NaCl was similar. However, since WH2 was a slight halophile, better growth of WH2 at 2.5% NaCl led to higher degradation rates. Thus, it can be inferred that when NaCl concentrations are 7.5% or lower, the degradation rate is not affected by NaCl concentration but is instead related to the growth of WH2.

### 3.7. MR Degradation Products Analysis

**Figure 5:**
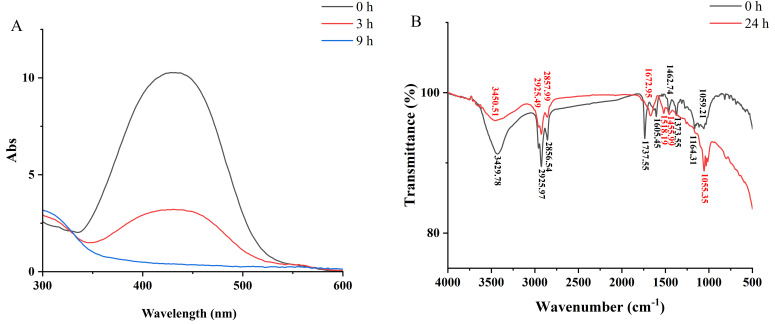
UV-vis and FTIR analysis of methyl red and its degradation products. (**A**) The UV-vis spectral analysis of MR treated by WH2 for different times. (**B**) FTIR analysis of methyl red and its degradation products. 0 h: Methyl red; 24 h: Degradation products of methyl red treated by WH2 for 24 h.

The degradation activity of WH2 on MR was analyzed by UV-visible scanning spectroscopy. As shown in Figure 5A, methyl red has a maximum absorption peak at 428 nm. Comparing with the initial absorption peak at 428 nm, the absorption peak of MR decreased after the treatment by WH2 for 3 h and completely disappeared after the treatment by WH2 for 9 h, indicating that MR was almost completely degraded by WH2. Therefore, it was proved that *Klebsiella aerogenes* WH2 had the ability to degrade MR and caused changes in the molecular structure of the MR dye. Meanwhile, an extra absorbance peak (300–350 nm) appeared after 3 and 9 h of treatment by WH2. This absorbance peak might be due to the new degradation products generated from MR degradation [40]. The result of the UV-vis spectrum confirmed that the characteristic absorption peak of MR decreased under the treatment of WH2 (Figure 5A).

FTIR analysis was conducted to further demonstrate the possible degradation products. In the FTIR analysis of MR (Figure 5B), the absorption peak around 3429.78 cm^−1^ represented the amines N-H stretch [24]. It was found that this peak shifted to 3450.51 cm^−1^ after 24 h of bacterial degradation, which might be attributed to the free N-H stretching vibration in the degradation product of MR, such as N,N-dimethyl-p-phenylenediamine. The absorption peaks at 2925.97 cm^−1^ and 2856.54 cm^−1^ were attributed to the asymmetric and symmetric stretching vibrations of -CH2-, respectively [18]. A decrease in intensity was observed for the C-H stretching vibrations in the characteristic peaks at 2925.49 cm^−1^ and 2857.99 cm^−1^ after 24 h of bacterial degradation, indicating that the molecular structure has undergone partial degradation. The absorption peak around 1605.45 cm^−1^ represented the vibration of the absorption peak of the azo double bond (–N=N) [25]. After 24 h of bacterial treatment, the absorption peak of the azo double bond (–N=N) was no longer visible, indicating that the azo bond of MR was reduced and the dye was degraded by bacteria. The peaks originally located at 1164 cm^−1^ and 1059 cm^−1^ (associated with C-N stretching) merge into a single, significantly deepened (intensified) peak at 1055 cm^−1^ after 24 h of bacterial treatment. The convergence of these bands into one intensified peak at 1055 cm^−1^ is characteristic of the formation of primary or secondary aromatic amines [41]. Comparison of the FTIR spectra of MR before and after treatment by WH2 revealed that certain peaks disappeared and new peaks emerged, which indicated that the dye had transformed to some other compounds or metabolites. Therefore, the results of FTIR analysis showed the disappearance of the azo bonds, which also indicated the degradation of the MR dye by WH2.

**Figure 6:**
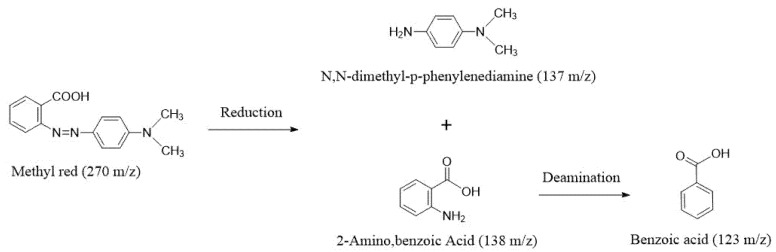
Prediction pathway of MR biodegradation by *Klebsiella aerogenes* WH2.

LC-MS analysis was conducted to investigate the metabolites and the possible degradation pathway of MR dye after the treatment of WH2 (Figure 6, Table S1). Control (methyl red) exhibited one peak corresponding to MR at a retention time of 7.97 min. MR degradation metabolites by WH2 indicated that two possible degradation products, N,N-dimethyl-p-phenylenediamine (DMPD, 137 *m*/*z*) and 2-amino, benzoic acid (2-ABA, 138 *m*/*z*), were formed initially during reductive cleavage of an azo bond. Subsequently, 2-amino, benzoic acid was possibly oxidized to benzoic acid. Similar results regarding MR degradation by other bacteria have also been reported [24,30,42]. However, further research is needed on the MR degradation pathways, except for its three degradation products: DMPD, 2-ABA, and benzoic acid.

### 3.8. Toxicity Studies

The environmental toxicity of MR degradation products was evaluated on seed germination and the length of root, shoot, and leaves of mung beans (*Vigna radiata* L.). The MR degradation products were diluted 1:2 and subsequently used for the phytotoxicity experiment. As shown in Table 1 and Figure S2, there was 100% seed germination in the presence of WH2-treated and untreated MR dye solutions. There was no significant difference in the germination rate of mung beans among the three groups. The length of the roots and stems of the MR-treated group and the degradation product-treated group was significantly shorter than that of the control group, respectively. The length of the stems and leaves of the degradation product-treated group was significantly longer than that of the MR-treated group, respectively. Thus, the toxicity of detoxification MR products was lower than that of the original MR. Previous studies have shown that two MR degradation products, DMPD and 2-ABA, do not exhibit toxicity towards *Sorghum vulgare*, *Phaseolus mungo*, and *Vigna radiata* [11]. Therefore, the degradation of the MR by WH2 significantly reduces its biotoxicity.

### 3.9. Integrated Discussion

In this study, the decolorizing activity of the azo dye methyl red (MR) by the newly isolated *Klebsiella aerogenes* WH2 (WH2) was evaluated. WH2 was able to decolorize 86.0% of MR at concentrations of 300 mg/L within 24 h. Currently, most pure microbial strains can only degrade MR at concentrations below 100 mg/L, whereas only a few strains can degrade MR at concentrations above 100 mg/L. For example, *Schizophyllum commune* 15R-5-F01, *Sphingomonas paucimobilis*, and *Kocuria indica* DP-K7 showed high decolorization efficiency for MR in the concentration of 100 mg/L, 750 ppm, and 200 μM, respectively [11,12,13]. Therefore, WH2 exhibited a relatively high level of degradation capacity for MR.

The degradation capacity and efficiency of the pure strain depend on its activity and adaptability. Generally, pure microbial cells rarely possess both high degradation efficiency and strong tolerance simultaneously. *Schizophyllum commune* 15R-5-F01 could degrade over 96% of MR (100 mg/L) within 3 h. It performs optimally at moderate temperatures around 30 °C, and salinities below 5%. *Sphingomonas paucimobilis* decolorized 99.63% of MR (750 ppm) within 10 h at 30 °C and pH 9. *Zhihengliuella* sp. ISTPL4 Strain showed 98.87% of 500 mg/L methyl red dye degradation within 12 h. The optimum temperature for decolorization of *Sphingomonas paucimobilis* and *Zhihengliuella* sp. ISTPL4 was found to be 30 °C [24]. The optimal degradation temperature for WH2 was 45 °C, and it can tolerate 10% NaCl as well as various metal ions, such as Ca^2+^, Fe^2+^, Cd^2+^, Mg^2+^, Mn^2+^, and Cu^2+^. Due to its good degradation ability and tolerance to 45 °C, salt concentration, and metal ions, the strain WH2 shows great potential for application.

The practical application requires careful consideration of biosafety, regulatory compliance, and ecological impact. *Klebsiella aerogenes* is a Gram-negative opportunistic pathogen from the *Klebsiella* species and the *Enterobacteriaceae* family. Previous studies have shown that *Klebsiella aerogenes* also exhibited degradation abilities against lignin and other azo dyes such as Acid Orange, Methyl Orange, Congo Red, Direct Blue 71, Direct Green 28, and Malachite Green, giving it significant application potential [43,44,45]. Nevertheless, its biosafety and ecological impact must be taken into account in future real-world applications.

The salts present in textile effluents—including sodium chloride, sodium carbonate, sodium hydrogen carbonate, and sodium phosphate—are used in dyeing and printing to improve dye fixation on fabrics. Sodium chloride is commonly used by researchers to simulate such effluents [46]. Kędzierska-Sar et al. used a NaCl concentration of 75 g/L (7.5%) to simulate high-salinity conditions in real textile wastewater [47]. WH2 retained its azoreductase activity in the presence of 7.5% NaCl, enabling its potential application for MR degradation in real-world saline wastewater. This study only examined the decolorization and degradation efficiency of a single dye (MR) by WH2 in the presence of NaCl. Hence, further studies are needed on mixtures of reactive dyes and actual dye manufacturing effluents to confirm efficacy for the actual dye wastewater.

## 4. Conclusions

In the current study, newly isolated *Klebsiella aerogenes* WH2 (WH2) can effectively decolorize 86.0% of methyl red (MR) at 300 mg/L in 24 h. As a halophile, WH2 retained its decolorization activity even in the presence of several metal ions and 10% NaCl. The azoreductase activity of WH2 can be maintained in the presence of NaCl at no higher than 7.5%. Furthermore, the results of FTIR and LC-MS revealed the possible degradation products of MR by WH2. Phytotoxicity analysis based on seed germination of *Vigna radiata* indicated that the degradation products of MR exhibited less toxicity than the parent compound. Although this study confirms WH2’s decolorization efficacy under controlled laboratory conditions, further investigation is required to assess its performance and stability in complex, real-world industrial effluents. Overall, the findings suggest that *Klebsiella aerogenes* WH2 holds significant promise for the future bioremediation of azo dye pollution in saline industrial wastewater from the textile industry.

## Figures and Tables

**Table 1:** Phytotoxicity of degradation products of MR on the seeds of *Vigna radiata* after 6 days of incubation.

Observations	*Vigna* *radiata* L. (*Mung beans*)		
	A	B	C
Root length (cm)	7.73 ± 5.60 ^a^	0.48 ± 0.17 ^b^	0.96 ± 0.31 ^b^
Stem length (cm)	10.74 ± 2.81 ^a^	0.95 ± 0.25 ^c^	2.33 ± 0.61 ^b^
Leaves length (cm)	1.65 ± 0.39 ^a^	0.67 ± 0.15 ^c^	1.29 ± 0.32 ^b^
Germination (%)	100 ^a^	100 ^a^	100 ^a^

A. Control: Seeds germinated in distilled water; B. Methyl red: Seeds germinated in methyl red (100 mg/L) for 6 days; C. Degradation products of methyl red: Seeds germinated in degradation products of methyl red (100 mg/L) treated by WH2 for 6 days. Data are presented as mean ± SD from three biological replicates. Different superscript letters (a, b, c) within the same row indicate significant differences among different treatment groups (*p* < 0.05).

## Data Availability

The original contributions and data presented in this study are included in the article.

## Supplementary Materials

The following supporting information can be downloaded at: https://www.mdpi.com/article/10.3390/microorganisms14040864/s1. Figure S1. The effect of methyl red (200 mg/L) on the growth of WH2 in MI and MII medium. A corresponding medium control was included. MII medium and MI medium were control cultures containing the same medium without methyl red. Figure S2. Phytotoxicity study of *Vigna radiata* L. which were treated by methyl red and its decolorization products for 6 days. Control indicated distilled water; Degradation products of Methyl Red indicated the decolorized products of Methyl Red treated by WH2; Methyl Red indicated untreated methyl red. Table S1. The GC-MS analysis of methyl red products obtained using different treatments.
