# Supplementary material for: Biodecolorization and Biodegradation of Methyl Red by Halophilic Klebsiella aerogenes WH2
Source: Microorganisms. 2026 Apr 11;14(4):864. doi: 10.3390/microorganisms14040864 (PMC13118744; doi:10.3390/microorganisms14040864)
Supplement: Supplementary file 1 [file microorganisms-14-00864-s001.zip › microorganisms-4221581-supplementary.pdf]

Supplementary material

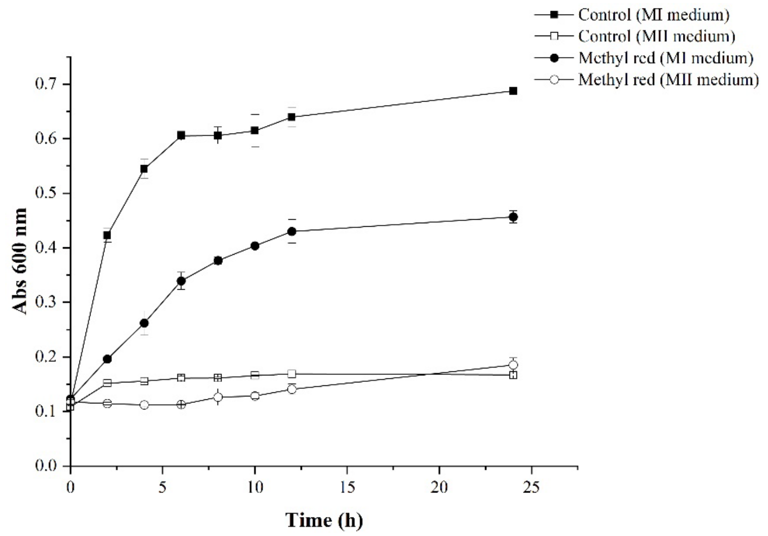

Figure S1 The effect of methyl red (200 mg/L) on the growth of WH2 in MI and MII medium. A corresponding medium control was included. MII medium and MI medium were control cultures containing the same medium without methyl red.

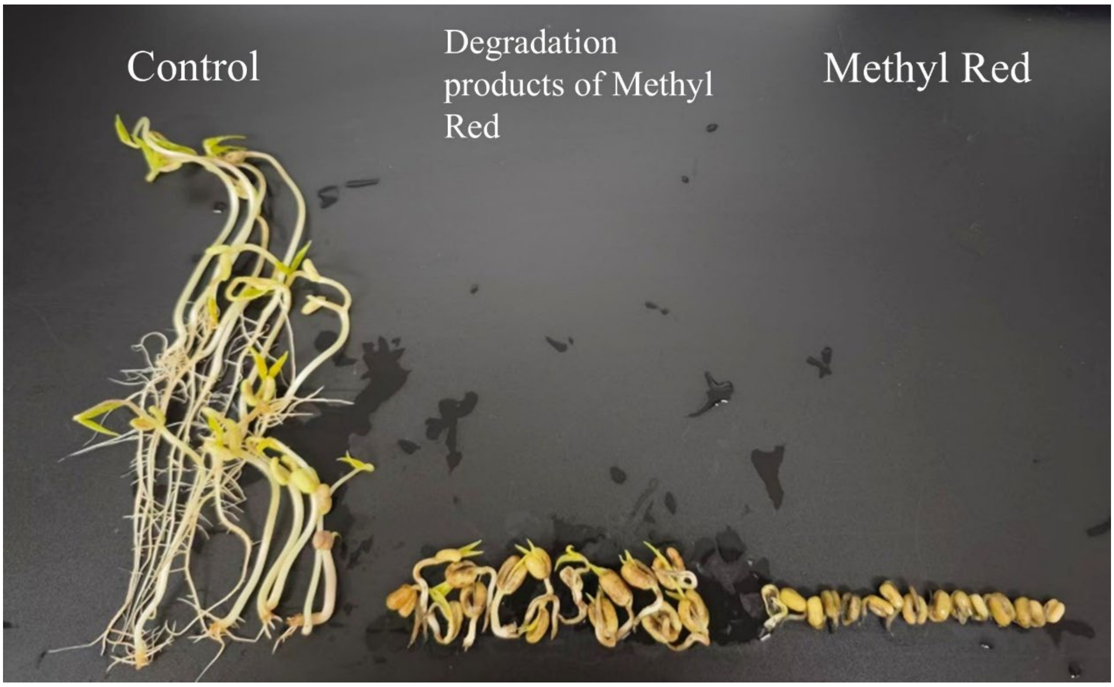

Figure S2 Phytotoxicity study of *Vigna radiata* L. which were treated by methyl red and its decolorization products for 6 days.
Control indicated distilled water;
Degradation products of Methyl Red indicated the decolorized products of Methyl Red treated by WH2;
Methyl Red indicated untreated methyl red.

Table S1. The GC-MS analysis of methyl red products obtained using different treatments.

ID	Rt. time (min)	MW and m/z	Name of product	Mass spectrum
[I]	7.97	MW-169<br>m/z-170 (+1)	Methyl red	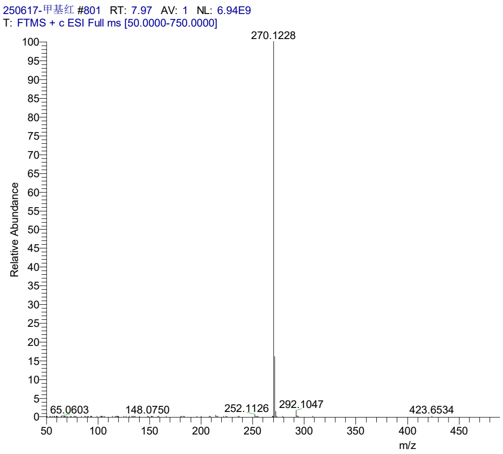
[I]	5.89	MW-137<br>m/z-138 (+1)	2-Amino, benzoic Acid	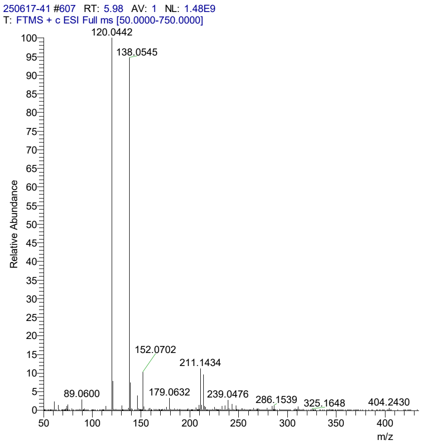
[II]	1.04	MW-136<br>m/z-137 (+1)	N,N-dimethyl-p-phenylenediamine	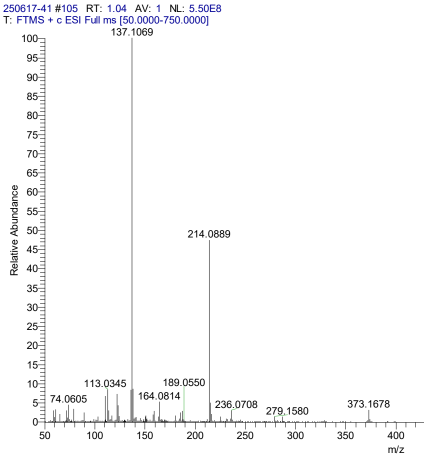

[III]	5.92	MW-137<br>m/z-136 (-1)	4-Aminobenzoic acid	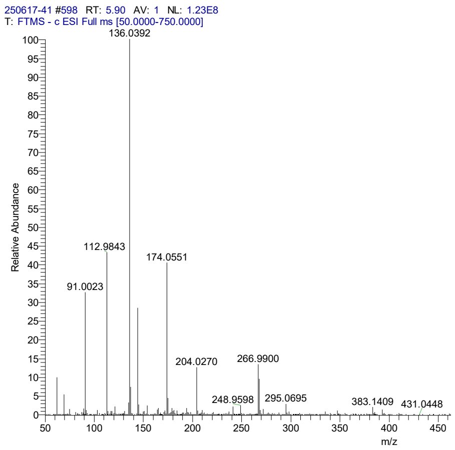
[IV]	0.92	MW-122<br>m/z-123 (+1)	Benzoic acid	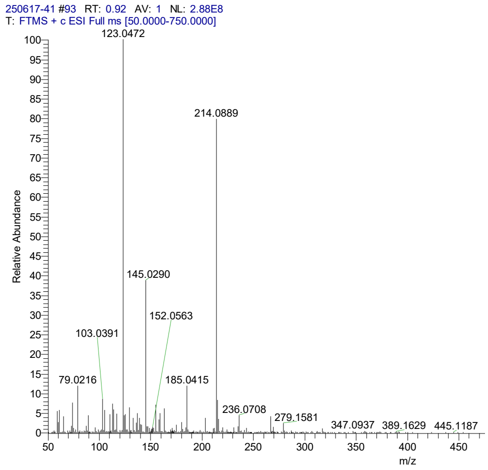
